# The Effect of Continuous Carbon Fiber Reinforcement on 3D-Printed Honeycomb and Re-Entrant Sandwich Panels Subjected to In-Plane Compression

**DOI:** 10.3390/ma18245594

**Published:** 2025-12-12

**Authors:** Andrei Nenciu, Dragoş Alexandru Apostol, Dan Mihai Constantinescu

**Affiliations:** 1 INCAS—National Institute for Aerospace Research “Elie Carafoli”, Bulevardul Iuliu Maniu 220, 061136 Bucharest, Romania; nenciu.andrei@incas.ro; 2 Department of Strength of Materials, National University of Science and Technology POLITEHNICA Bucharest, Splaiul Independenţei 313, 060042 Bucharest, Romania; dragos.apostol@upb.ro; 3 Institute of Solid Mechanics of the Romanian Academy, Str. Constantin Mille 15, 010141 Bucharest, Romania; 4 Technical Sciences Academy of Romania, Bulevardul Dacia 26, 030167 Bucharest, Romania

**Keywords:** printed sandwich panels, honeycomb, re-entrant, continuous carbon fiber, in-plane compression, digital image correlation, energy absorption

## Abstract

This study examines the in-plane compression behavior of sandwich panels produced with additive manufacturing. This study focuses on two types of honeycomb unit cell topologies with larger dimensions: a hexagonal one and a re-entrant one. For each panel geometry, two material configurations were examined: Onyx (a nylon-based composite) and Onyx reinforced with 10% continuous carbon fibers (CCFs) by mass. The objective was to assess the influence of fiber reinforcement on the mechanical performance and deformation response of the panel structures. In-plane compression tests were conducted to determine the stiffness, strength, and failure modes of the specimens. Additionally, the digital image correlation (DIC) technique was used to capture full-field strain distributions and analyze local deformation mechanisms during loading. The results revealed distinct mechanical responses between the two geometries: the re-entrant structure exhibited auxetic behavior and enhanced energy absorption characteristics. Although reinforced honeycomb panels have an average load capacity that is 35% higher, they fail at a displacement that is approximately 55% smaller compared to unreinforced panels. Despite accounting for only 25% of the total number of layers and 10% of the panel’s mass, the reinforcement achieved superior strength. Re-entrant panel testing showed a 25% force increase in favor of the reinforced variant. They fail at a displacement that is 36.5% greater than that of reinforced honeycombs. This demonstrates a more compliant response while also maintaining 4.9% greater strength, indicating the superior behavior of auxetic reinforced sandwich panels. Introducing CCF reinforcement increased the load-bearing capacity and reduced localized strain concentrations without altering the overall deformation pattern. These findings suggest that enhancing materials can increase the strength and flexibility of 3D-printed re-entrant structures, providing valuable insights for lightweight design and optimized material use in structural applications.

## 1. Introduction

Sandwich structures have garnered significant attention in engineering fields that require components to be both lightweight and mechanically robust, particularly in aerospace [1], automotive [2], and defense industries [3]. The mechanical efficiency of sandwich panels is primarily influenced by the geometry of the core, the properties of the constituent materials, and the quality of the manufacturing process. Traditionally, honeycomb cores were made using metallic or polymer sheets that were bonded through conventional methods. However, the advent of additive manufacturing (AM) technologies has enabled the design and fabrication of complex cellular architectures, resulting in customized mechanical properties and enhanced structural functionality [4,5].

Among various core designs, the hexagonal honeycomb structure is renowned for its exceptional stiffness-to-weight ratio, high compressive strength, and effective energy absorption characteristics [6]. In contrast, the re-entrant honeycomb structure features an auxetic topology, exhibiting a negative Poisson’s ratio. This unique property grants it superior shear resistance, indentation toughness, and recoverability under compressive deformation [7]. The re-entrant honeycomb core demonstrates improved shear properties due to a snap-through instability, significantly enhancing its energy absorption capability compared to conventional materials. The comparison between conventional and auxetic honeycomb cores remains a prominent area of research [8,9,10,11], especially when these structures are fabricated using polymer-based composite materials through AM technologies. Continuous fiber can be used to reinforce the faces [8], while lightweight customized lattice structures could be selected for the core, all built integrally in one single process. Unfortunately, additive manufacturing of continuous fiber-reinforced polymer composites faces reliability challenges in achieving consistent flexural strength and stiffness. If the production replicated real-world conditions, including filament spool changes, fiber aging, and time gaps between batches, it was found that the mechanical properties were consistent in early batches [12], but variability in flexural strength and stiffness increased from one batch to the next, reaching deviations up to 70% for carbon fiber in later batches. A further development led to bio-inspired fractal structures through three fundamental shape units (curve, circle, and hexagon), which were constructed and investigated as having distinct structure ratios related to cell sizes at different geometry levels [13].

Reference [14] provides a thorough review of recent advancements in additively manufactured materials and lightweight structures, focusing on their mechanical properties, particularly in energy absorption applications. The review covers a variety of design optimization techniques, including parametric optimization, topology optimization, and stochastic optimization, all while considering uncertainties introduced during fabrication. Moreover, it highlights the significant potential of data-driven and machine learning approaches in additive manufacturing for managing process–property relationships and for in situ monitoring. Other review examinations of the advancements and state-of-the-art developments in continuous carbon fiber (CCF)-reinforced thermoplastic composite materials, focusing on their processing and fabrication through the material extrusion method, were presented recently in [15,16]. These studies explore how varying printing process parameters can influence the overall mechanical performance of the produced composites. The development of innovative cellular structures incorporating continuous fiber, alongside an analysis of fracture mechanics within these materials, is also presented.

Two very recent reviews [17,18] examine recent advancements in 3D printing of short fiber-reinforced polymer composites and continuous fiber-reinforced polymer composites, emphasizing their potential to transform industrial applications. The evolution of additive manufacturing methods; material innovations (including bio-based polymers, recycled fibers, and nanofiber-reinforced systems); and their impact on tensile, shear, flexural, toughness, and energy absorption behavior is presented. Insights into the current state of continuous fiber cellular structures, focusing on the structural design methodologies and optimizations that underpin their development, applications across different industries, and the technological challenges that must be addressed, are comprehensively detailed.

However, the influence of printing parameters, especially the position of the carbon fiber layer on such material, has focused on optimizing different printing and testing parameters such as carbon fiber layer position, infill density, fiber angle, and strain rate in 3D-printed carbon fiber-reinforced nylon composite [19]. The optimal combination of these parameters is also recommended for maximizing the mechanical strength and energy absorption of related 3D-printed parts.

As shown in [20], optimization cases for the deposition of long carbon fiber-reinforced composites have been developed by enlarging raster height and adjusting nozzle feeding angles to mitigate substantial mechanical forces on fibers. Not to be neglected is the understanding of the dynamic temperature field and warpage generation during the printing of continuous carbon fiber thermoplastic composites. The mechanism of warpage deformation was uncovered to guide the optimization of AM process for the warpage mitigation in the as-built components by obtaining simulated data and experimental results [21]. Special efforts are dedicated to weight reduction and enhanced performance by using combined materials [22]. The Airbus H-160 helicopter utilizes carbon fiber-reinforced PEEK (polyetheretherketone) thermoplastic composites to replace titanium alloy in the rotor hub central part, leading to cost savings in manufacturing, improved structural damage tolerance, and enhanced maintainability. Very recently, a novel fiber-reinforced composite was constructed for the first time via a continuous printing procedure with flexible yet robust carbon nanotube (CNT) fibers as host [23]. In contrast to traditional carbon fiber, CNT fiber features a remarkable superiority in curvature radius at corners during continuous 3D printing, thereby endowing printed CNT fiber composites with a low porosity of only 8.11% and a high axial tensile strength of 674 MPa. Thus, new insights for developing a lightweight but high-strength printable filament are opened.

Onyx, a nylon-based composite filled with short carbon fibers, offers improved stiffness and surface finish. Additionally, the inclusion of CCF further increases the load-bearing capacity and stiffness of the printed parts. The influence of carbon fiber reinforcement orientation on the mechanical performance of Onyx FR-A composites, manufactured using the Markforged FX20 printer [24], is studied through mechanical tests, including tensile, compressive, open-hole tension, and interlaminar shear stress. The findings demonstrate that fiber orientation plays a crucial role in determining material behavior. The flexural performance of 3D-printed continuous fiber-reinforced composites focuses on the influence of fiber types, orientation, and temperature [25]. Using carbon, glass, kevlar fiber, and Onyx matrix filaments, three-point bending tests were conducted under different temperatures. The results reveal a significant influence of fiber type and orientation. Clearly, the printing process is susceptible to a variety of defects that are derived from the fabrication process parameters, such as porosity, insufficient fiber impregnation with the polymer, and fiber disorientation. The accurate detection and quantification of them is a crucial part of it, as studied experimentally by implementing an X-ray computed tomography testing campaign [26]. All properties depend on specific printing parameters and environmental conditions. The influence of two printing parameters, namely, the orientation and positioning of the parts on the printing platform and the influence of humidity on the mechanical properties of the parts, is studied on samples which were fabricated with onyx using a Markforged X7 printer [27]. The results showed that onyx could be considered an isotropic material to a certain extent because its mechanical properties do not vary sufficiently according to the orientation angle on the printing platform. The study of the sensitivity to humidity revealed that a specimen absorbs approximately 2% of the humidity and loses up to 65% of its Young’s modulus after 165 days of exposure, significantly influencing the mechanical properties of the parts. Consideration should be given to the aging of onyx when using printed parts as structural components.

For specific drone applications, the composite part is supposed to have high specific strength and rigidity. High-temperature Polyamide 6, continuous glass fiber-reinforced Onyx, and carbon fiber-reinforced Onyx composites are characterized for their mechanical and fracture behavior [28]. Their study provides a direction for the next generation of drone manufacturers.

Despite the growing body of research on polymeric sandwich cores, the combined effects of core geometry—such as hexagonal versus re-entrant—and material composition, specifically Onyx versus Onyx reinforced with continuous carbon fibers (Onyx + CCF), on the compressive behavior of additively manufactured sandwich panels have not been thoroughly investigated. This study assesses the benefits of using a re-entrant topology versus traditional honeycomb in terms of strength, flexibility, and energy absorption.

Quasi-static compression tests were conducted to evaluate the effect of core topology and reinforcement on the mechanical performance of the panel structures. Digital image correlation (DIC) was used to illustrate the distinct mechanical behaviors of the two configurations by analyzing the strain fields. Notably, the re-entrant structure exhibited auxetic behavior and improved energy absorption characteristics. Introducing CCF reinforcement enhanced the load-bearing capacity and reduced localized strain concentration due to its stiffness. Ultimately, the reinforced re-entrant topology demonstrated superior energy absorption capabilities.

## 2. Materials and Methods

### 2.1. Topology of Hexagonal Geometries

**Figure 1:**
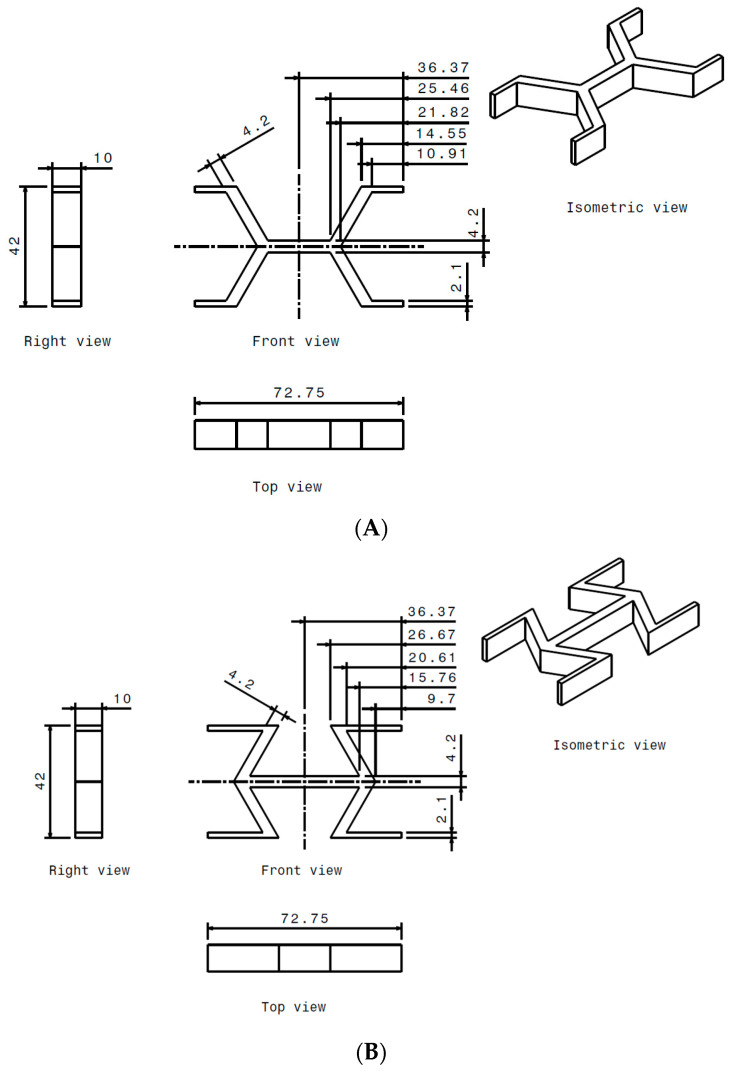
Geometric characteristics of the representative honeycomb volume: (**A**) hexagonal; (**B**) re-entrant. Overall dimensions of the volumes are the same. The cell angle changes from +57° to −57°.

Figure 1 illustrates the geometric characteristics of the two selected cell designs: the hexagonal cell (HCB) and the re-entrant cell (REN). To simplify the design process for the representative volumes, these cell geometries were created using a different symmetry plane than is typically found in the specialized literature. This method ultimately resulted in the well-known cells commonly used in panel design. The representative volume is the same for both topologies: 72.75 mm length, 42 mm height, and 10 mm thickness.

**Figure 2:**
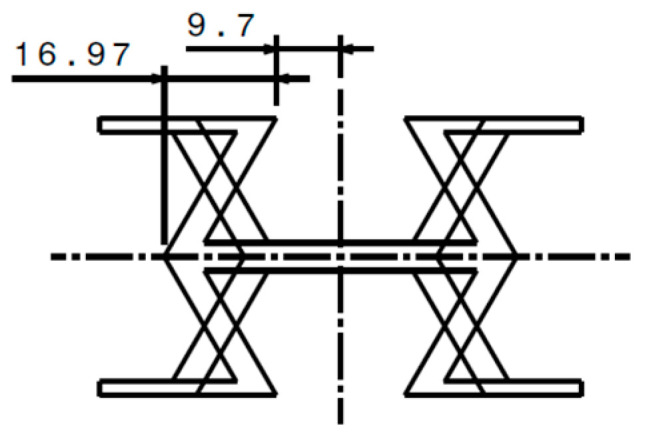
Comparison of overlapping HCB and REN unit cells.

To maintain the geometric characteristics of the HCB and REN cells, the REN cell was designed by mirroring the 16.97 mm section shown in Figure 2. This approach ensures that the geometry of the REN cell preserves all the specific features of the HCB cell, including angles, thicknesses, and wall positions.

Table 1 presents the properties of the representative volumes used in the tested sandwich panels, including mass, cell surface area, and relative density. The surface areas of the cells vary depending on their geometries. The HCB cell has a surface area of 580 mm^2^, while the REN cell has a larger surface area of 760 mm^2^, indicating a 31% increase. The relative density of the cells represents the degree of overlap between the cell structure surface area and the total area in which they are inscribed. The hexagonal cell (HCB) has a relative density of 0.189, while the re-entrant cell (REN) has a relative density of 0.248, making it 31.2% denser than the hexagonal cell. The mass to cell surface ratio, measured in grams per square millimeter (g/mm^2^), is a critical indicator of the performance of these representative volumes. Both the hexagonal honeycomb cells and the re-entrant cells exhibit the same efficiency, which is valued at 0.012 g/mm^2^, making them equivalent in this regard.

### 2.2. Materials

The first batch of additively manufactured panels is made from unreinforced material known as Onyx (Markforged, Waltham, MA, USA). This premium 3D printing material combines strength, stiffness, and stability, thanks to the carbon microfibers integrated into the nylon matrix. According to the manufacturer, Onyx is 1.4 times stiffer than ABS, making it an ideal choice for creating lightweight yet strong components. Furthermore, Onyx properties can be enhanced with continuous fiber filament to improve its strength even more. Detailed properties of Onyx are listed in Table 2, while the characteristics of the continuous fiber (CCF) material, as provided by the manufacturer, are shown in Table 3.

### 2.3. Printing of Sandwich Panels

The sandwich panels were produced using a Markforged X7 3D printer (Markforged, Waltham, MA, USA) through the continuous filament fabrication (CFF) process. The core material utilized was Onyx, a nylon-based composite reinforced with short carbon fibers, supplied as a 1.75 mm diameter filament. In the reinforced configuration, continuous carbon fiber was added during the printing process using a second printing head, which utilized a 0.35 mm diameter filament to strengthen selected layers within the structure. Print speed and temperature settings were automatically determined by the slicer software, with no possibility for external modification. Visual quality control was performed during and after printing, and no imperfections were observed; the measured panel dimensions were consistent with the technical drawings. A 100% solid infill pattern was used to ensure uniform material distribution and structural integrity.

**Figure 3:**
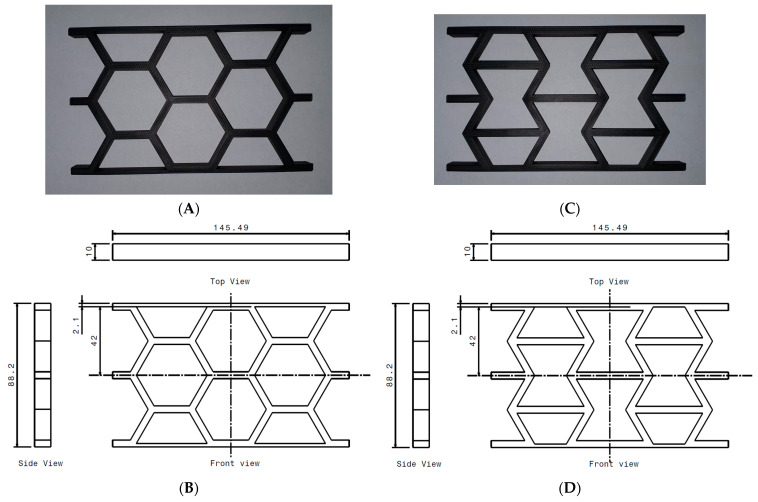
Additively manufactured panels: (**A**) panel with hexagonal cell; (**B**) dimensions of the panel with HCB cell; (**C**) panel with re-entrant cell; (**D**) dimensions of the panel with REN cell.

The geometry data of the unit cells has already been provided. Consequently, the technical drawings of the complete panels, as shown in Figure 3, only display the overall dimensions, specifically the length and height of the panels, including the added face sheets on the top and bottom. For each panel type, 8 panels were printed, as HCB and REN, and unreinforced and reinforced with CCF, with a total of 32 panels.

Additionally, it is important to note that the face sheets were added to the panels after the representative volumes were translated to create the complete parts. These sheets are essential for applying the compressive force to the panels.

The primary distinction between the two types of panels is the use of continuous carbon fiber filaments within their structures. Each panel has a thickness of 10 mm, comprising a total of 80 layers. The software allows users to select specific layers for reinforcement, providing a significant level of customization for part modifications. To prevent the introduction of asymmetric stiffness—which could cause undesirable mechanical behavior, such as bending—reinforcement is concentrated in the central layers of the panels. This strategy ensures that the reinforced area is distributed uniformly throughout the panels.

Table 4 presents data obtained from the slicer software for each type of panel. It is important to note that additively manufactured panels may not all have the same mass due to minor variations. Table 5 displays the actual weighted mass of each printed panel, highlighting differences among the 3D-printed panels, which are generally lighter than the theoretical values predicted by the slicer. For honeycomb core (HCB) panels, the actual mean mass is 4.83% lower, while for reinforced (REN) panels, the mass variation is 7.23%. This comparison indicates that the honeycomb panels align more closely with the values predicted by the slicer.

For unreinforced parts, users have the flexibility to choose the printing layer thickness. However, for reinforced parts, the layer thickness is dictated by the thickness of the continuous fiber filament used. To ensure consistency in printing parameters, it is important to verify the layer thickness set when carbon fiber is selected as the reinforcement method. When carbon fiber is chosen, the software automatically adjusts the layer thickness to 0.125 mm. Therefore, to maintain uniformity in the production of all parts, the selected layer thickness will be 0.125 mm for both unreinforced and reinforced batches.

As mentioned previously, the panels have a thickness of 10 mm. To reduce carbon fiber consumption and cost, the reinforced layers were situated only in the middle of the specimen through the thickness over the whole surface of the panel. The first layer begins at 3.75 mm from the surface, and consecutive layers extend up to 6.25 mm, with a total of 80 layers. This means that layers 31 to 50 are reinforced.

Table 6 shows the data collected from the slicer for each type of reinforced panel, which is similar to the values recorded for the unreinforced panels.

In Table 7, the average values of the weighted panels show slight differences compared to those in Table 5. The deviation between the two tables is consistent, with an average lower difference of 6.32% for honeycomb panels and 6.67% for re-entrant panels. These measurements indicate that although using filament for reinforcement may increase mass variation (for two panels out of eight compared to unreinforced panels), it also produces uniform results across the different additively manufactured panels.

**Figure 4:**
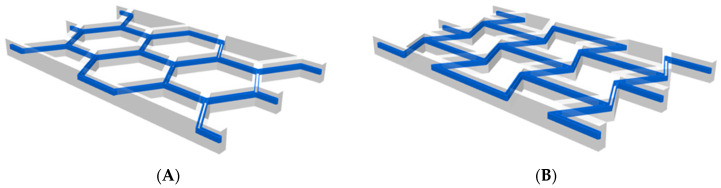
Distribution of continuous carbon fiber filament in panels: (**A**) hexagonal; (**B**) re-entrant.

Upon examining the fiber distribution in the panels, as presented in Table 6 and Table 7, it is clear that the REN panels utilize the highest amount of reinforced material in relation to the total volume of the panel. This indicates a more effective distribution of mechanical loads within the structure, although it also signifies a greater consumption of CCF. The distribution of the continuous carbon filament for both topologies is depicted in Figure 4.

**Figure 5:**
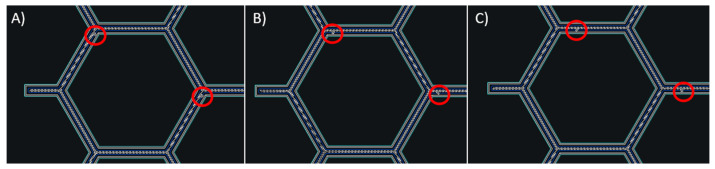
Starting point offset application method of continuous carbon filament: (**A**) first layer; (**B**) second layer; (**C**) third layer. In each layer, the starting point is shifted to avoid the superposition of possible internal defects located in the same position.

During the additive manufacturing process, the slicer program implements offsets for the starting points of the continuous carbon fiber in each subsequent layer to minimize the risk of defects or weak spots in the produced parts. Each layer incorporates five different carbon fiber filaments distributed across the surface of the panel. This method ensures that interruptions in the filament printing do not lead to printing issues in the components leading to major defects. As illustrated in Figure 5, the starting point for the printing process varies for each layer as marked by the red dot.

**Figure 6:**
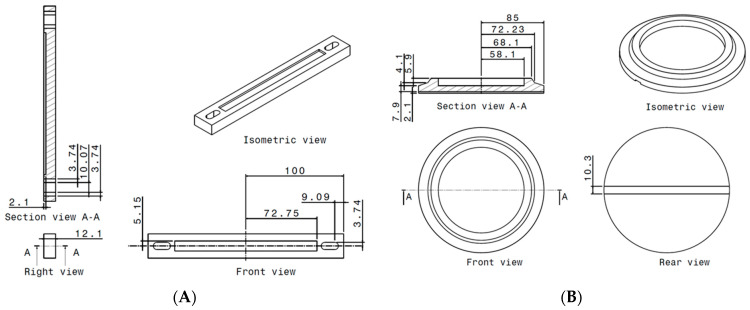
Additively manufactured auxiliary components: (**A**) lower fixing part; (**B**) upper fixing part.

To conduct compression tests on the panels, two auxiliary components were fabricated using additive manufacturing, as shown in Figure 6. These components were designed to securely hold and guide the specimens during testing, preventing any lateral movement.

### 2.4. Experimental Testing

The mechanical tests on the panels were carried out using the ZwickRoell (ZwickRuell, Ulm, Germany) Z010 universal testing machine. To analyze the detailed behavior of the panels during these tests, the digital image correlation (DIC) system, ZEISS ARAMIS 12 MP (Carl Zeiss GOM Metrology GmbH, Braunschweig, Germany) was utilized. A caliber CP40/170) was used for the measurements. The DIC analysis was performed using a facet size of 25 pixels and a facet step of 15 pixels, ensuring increased accuracy and overlap for evaluating the strain field. A mask has been placed in order to measure only the panel’s deformations. 

For the assessment of the components’ mechanical behavior, in-plane compression testing was performed at a controlled loading speed of 1 mm/min. Tests were performed at room temperature (21–22 °C) and humidity of 55–60%. Prior to testing, the panels were kept in a closed environment to avoid the influences of humidity.

This DIC method enables a thorough examination of the deformation, including possible crack initiation, local failure, and the emergence of potential instability zones. Additionally, it allows for the identification of any differences among the panels or unique structural features that may influence their response to in-plane compression.

During the test, each specimen is accurately positioned within the additively manufactured components of the testing machine. Both force and displacement are continuously recorded throughout the process. The results are then analyzed to evaluate the behavior of each panel and to identify any variations that may be related to geometry or manufacturing defects.

### 2.5. Finite Element Modelling

**Figure 7:**
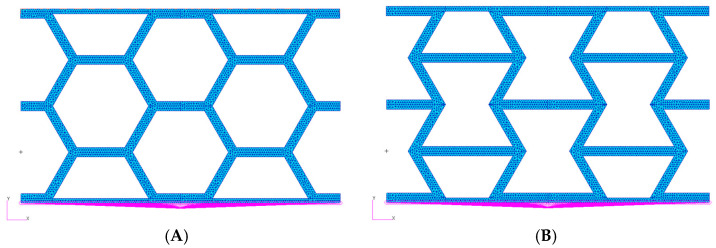
FEM model: (**A**) HCB panel; (**B**) REN panel.

To conduct the finite element simulations (FEM), we developed two distinct models: one for the hexagonal cell configuration and another for the re-entrant cell configuration, as shown in Figure 7. To accurately replicate the boundary conditions from the experimental tests, all translational degrees of freedom at the bottom of each model were constrained using an RBE2 rigid element. This rigid element was also used to extract the reaction force from the panel, facilitating direct comparison with the experimental results. At the top of the model, another rigid element was implemented to simulate the upper grip of the testing machine, to which a total displacement of 15 mm, identical to that applied during the physical tests, was imposed.

The hexagonal configuration model comprises 3578 nodes and 5616 elements, while the re-entrant configuration model includes 4342 nodes and 6936 elements.

The finite element models were developed using pre- and post-processing MSC.Patran 2017.0.2, and the solver MSC.Nastran 2017.0. A nonlinear solution was adopted. Materials were defined using MAT1 for Onyx and MAT8 for Onyx + CCF. The structural components were modeled with 2D thin shell elements (CTRIA3) with a uniform mesh size of 1 mm. Shell elements were selected to represent the 10 mm thick structures, enabling the development of a simplified equivalent model capable of capturing the global structural behavior while maintaining computational efficiency.

The finite element model was formulated as a simplified representation using homogenized panel properties derived from experimental tests. This approach excluded an explicit representation of the CCF + Onyx interface, interlayer bonding, porosity, and other microstructural imperfections. While this simplification allowed for efficient computation and accurate prediction of global stiffness and deformation for Onyx-only panels, it introduced limitations when applied to reinforced panels (Onyx + CCF). Specifically, the model overestimates reaction forces in these multi-material panels due to its inability to capture complex interfacial interactions, local delamination, and other failure mechanisms.

The analysis was performed as a quasi-static nonlinear simulation using 30 load increments, with a maximum of 20 iterations allowed per increment. Convergence criteria were set at 1% for both displacement and load errors to ensure reliable prediction of the global structural response.

A mesh sensitivity study was not conducted because the primary objective was to evaluate global structural trends rather than localized phenomena, which typically require finer meshes.

Although solid elements would have been more suitable for investigating local effects such as interlaminar stresses, delamination initiation, and local buckling, future studies will address these phenomena highlighting areas for potential refinement.

For this study, the shell-based approach provides favorable numerical stability, reduces the number of degrees of freedom, and facilitates incorporating homogenized properties. This enables a practical, computationally efficient modelling framework.

The modelling assumptions and simplifications are justified because the finite element model effectively captures global structural behavior for single-material configurations but demonstrates limitations for multi-material panels, where the interactions between the constituent materials and interfaces are critical. Despite these limitations, the approach reliably and efficiently predicts overall panel behavior, consistent with established practices in the literature.

Prior to the FEM modeling, tensile tests were conducted on both material configurations, one for the base material (Onyx) and the other for the reinforced configuration with continuous carbon fiber (Onyx + CCF). These experimental results (presented in detail in [31]) allowed the panels to be modeled as homogeneous with accurately defined mechanical properties, as shown in Table 8.

**Figure 8:**
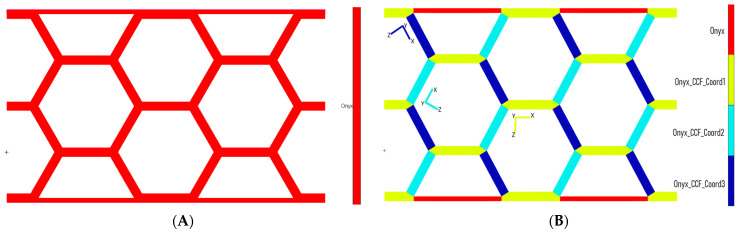
Properties definition for the two configurations: (**A**) Onyx, (**B**) Onyx + CCF.

For the unreinforced panels, the material was assumed to be isotropic. In contrast, for the reinforced configurations, the carbon fibers were oriented parallel to the edges of the cells. This necessitated the definition of multiple local coordinate systems, which allowed for the assignment of material properties relative to these systems to accurately represent the distribution of the fibers. The reinforced material was modeled as 2D orthotropic, with its properties derived directly from experimental data. The failure criteria for both configurations were defined using the maximum stress criterion, based on the results of tensile tests. Figure 8 illustrates how the properties were defined for the two material configurations.

## 3. Results

Compression mechanical testing was conducted in two stages: first, according to the force–displacement data provided by the testing machine, and second, utilizing the digital image correlation (DIC) method. To present the results in a clear and organized manner, they are categorized into two groups: unreinforced panels (N) and reinforced panels (R). Each category includes both hexagonal (HCB) and re-entrant (REN) honeycomb panels.

### 3.1. Mechanical Tests Results

#### 3.1.1. Unreinforced Panels with Hexagonal Unit Cells

**Figure 9:**
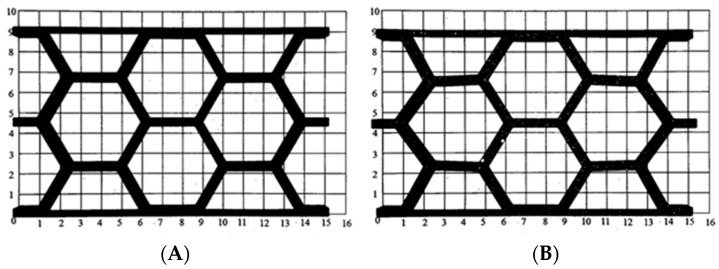
Unreinforced hexagonal panels: (**A**) before testing; (**B**) after testing and recovery. The scaled grid is in centimeters.

The unreinforced hexagonal honeycomb panels (HCB_N) demonstrated consistent performance, with the maximum force variation among the eight tested panels (labeled from 1 to 8 for tracking purposes) remaining within a range of 270 N. During the tests, the force–displacement curve showed a gradual deformation of the panels under compressive load. An unloaded HCB panel is shown in Figure 9A, as an example. After loading and unloading, the panels did not return to their original shape and exhibited some degree of plastic deformation; however, their recovery was remarkable (see Figure 9B).

The results presented in Table 9 indicate the maximum force values for each tested panel, along with the corresponding displacement at which these forces occur. For the HCB_N panels, the average force value is approximately 1071 N, with an average displacement of 9 mm.

**Figure 10:**
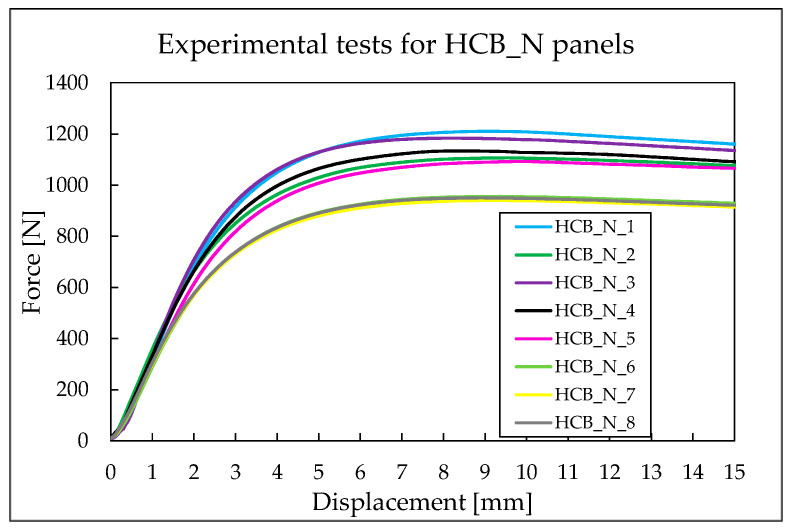
Force–displacement curves for HCB_N panels.

For the unreinforced hexagonal panels, the maximum recorded force is 1210.26 N, while the minimum force is 939.78 N. Although there is a difference of approximately 270 N between these two values, Figure 10 shows that the maximum force occurs at a similar displacement across all panels.

#### 3.1.2. Unreinforced Panels with Re-Entrant Unit Cells

The unreinforced re-entrant panels (REN_N) displayed a similar loading trend; however, there was approximately a 250 N variation in the maximum force among the tested panels. The behavior observed during the tests, particularly the force–displacement curve, indicates a failure mode similar to that of the HCB_N panels under compression load.

The maximum force values for each REN_N panel tested were recorded, along with the corresponding displacements at which these forces occurred. As shown in Table 10, the average force value is approximately 1250 N.

**Figure 11:**
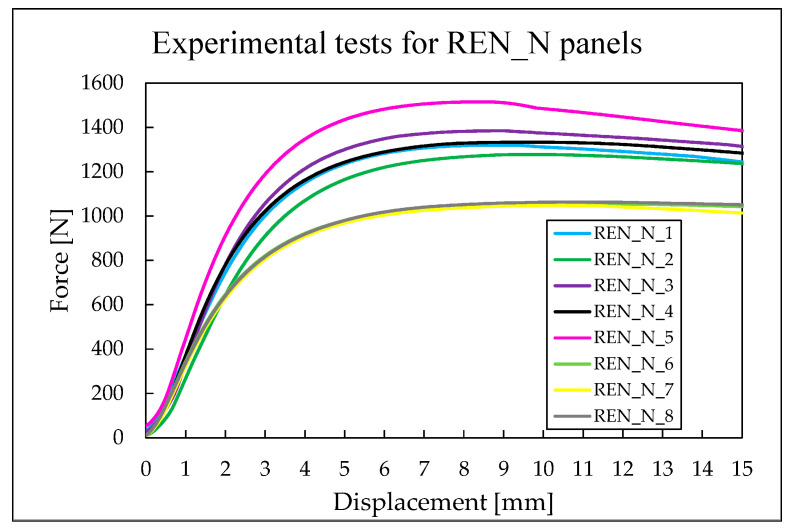
Force–displacement curves for REN_N panels.

The unreinforced re-entrant panels exhibit a maximum force value of 1514.15 N and a minimum value of 1048.48 N. The difference of approximately 466 N between these maximum and minimum values is illustrated in Figure 11. The displacement at which the panels fail ranges from 8.39 mm for the highest value of the maximum force to 10.82 mm.

#### 3.1.3. Reinforced Panels with Hexagonal Unit Cells

The reinforced hexagonal honeycomb panels (HCB_R) demonstrated consistent behavior; however, their response to compressive loading differed from that of the unreinforced panels. The failure characteristics, indicated by the force–displacement curve, show a sudden and steep collapse of the material, highlighting the influence of the carbon fiber reinforcement on the structure. After the load was applied, the panels failed to return to their original shape due to their greater stiffness, making them less compliant compared to the unreinforced panels.

Based on the results presented, the maximum force recorded for each HCB_R panel, along with the corresponding displacement at which it occurred, was determined and is detailed in Table 11. The panels reached an average maximum force of 1527 N.

**Figure 12:**
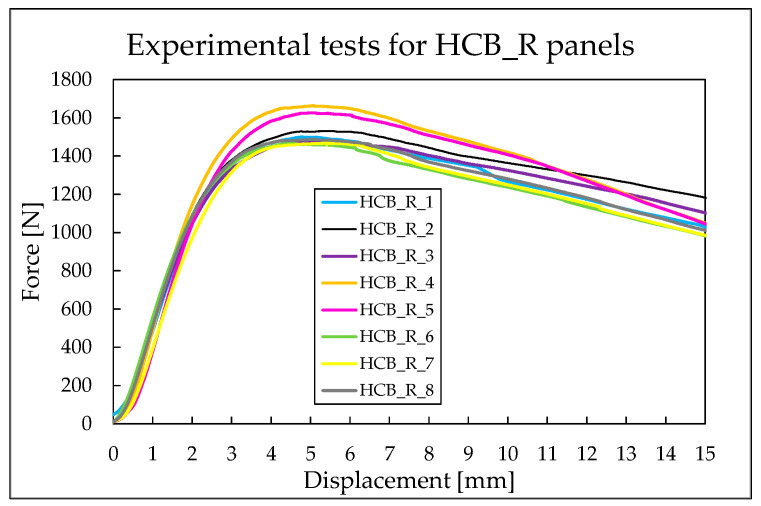
Force–displacement curves for HCB_R panels.

For the reinforced hexagonal honeycomb panels, the maximum force value recorded is 1663.15 N, while the minimum value is 1466.42 N. In the case of the HCB_R panels, the difference between the minimum and maximum force values is smaller—approximately 197 N—compared to 270 N for the HCB_N panels. Figure 12 shows that the displacement at which the maximum force occurs in the panels varies only between 4.59 mm and 5.48 mm, resulting in a range of just 0.88 mm. It is noteworthy that all eight curves for the reinforced panels stand closer together compared to the curves obtained for the unreinforced panels. This indicates that the panels with CCF exhibit a stiffer behavior, as expected.

#### 3.1.4. Reinforced Panels with Re-Entrant Unit Cells

The reinforced re-entrant panels (REN_R) displayed a similar uniform behavior to the unreinforced panels. Despite being reinforced, their failure occurred gradually, much like that of their unreinforced counterparts. The testing results, indicated by the force–displacement curve, demonstrate the influence of carbon fiber on the structure. However, because of the cell geometry, the failure in the REN_R panels was not as abrupt as that seen in the HCB_R panels.

Based on the results obtained, the maximum force and the corresponding displacement were recorded for each tested REN_R panel. As shown in Table 12, these panels achieved an average maximum force of 1603 N, which is greater than that of the HCB_R panels.

**Figure 13:**
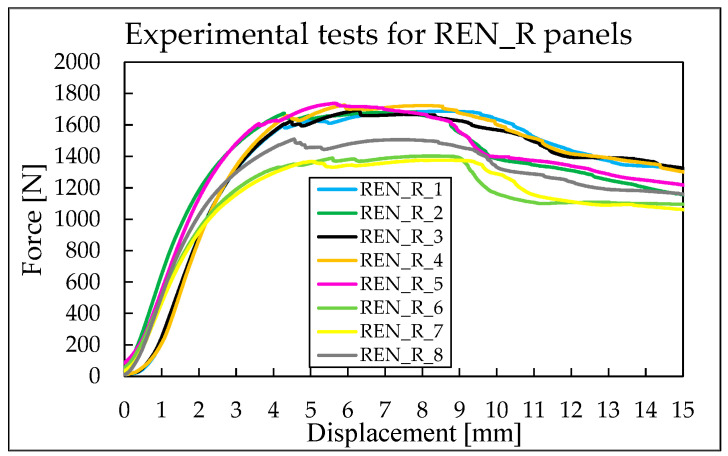
Force–displacement curves for REN_R panels.

For the REN_R panels, the maximum registered force is 1737.96 N, while the minimum value is 1376.35 N, resulting in a difference of about 362 N between these extreme values. As shown in Figure 13, the auxetic behavior of the cells is emphasized by the carbon reinforcement. The curves display some local instabilities, indicating a more pronounced re-arrangement of the cell geometry compared to the HCB_R case, continuing until the final imposed displacement. After reaching the maximum force, it remains relatively constant on a plateau before gradually decreasing.

### 3.2. Digital Image Correlation Results

To analyze the behavior of the panels in more detail, the digital image correlation (DIC) method was employed on several panels to investigate the deformation patterns. While this method is effective for obtaining global fields of strains and displacements, local values were extracted in 12 points to reduce the volume of analyzed data. For illustration, the results presented for each type of configuration are based on panel number 6 at the moment the maximum force was recorded.

#### 3.2.1. DIC for Hexagonal Honeycomb Panels

**Figure 14:**
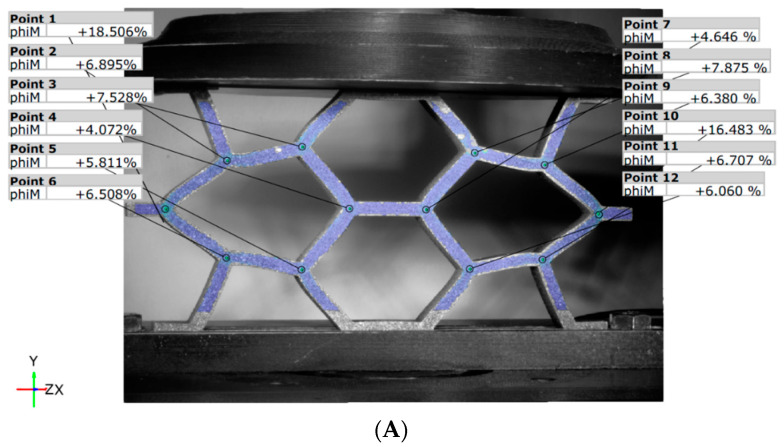

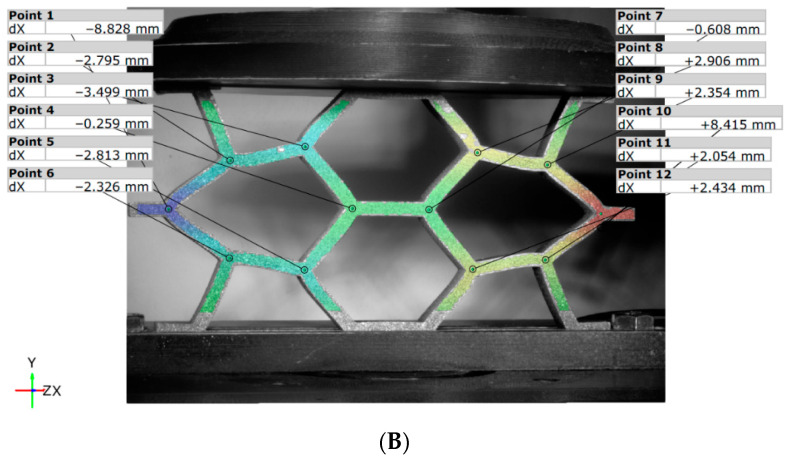
DIC results for the HCB_N panel: (**A**) von Mises strains; (**B**) displacements along X-axis.

Based on the DIC interpretation, it is evident that for the HCB_N type panel (see Figure 14), the strain values are fairly consistent across all points. However, points 1 and 10, located in the middle of the panel, exhibit significantly higher strain values of approximately 18.5% and 16.5%, respectively. The displacement pattern along the X direction is as expected, with these same points moving away from the median Y-axis of symmetry by about 8.8 mm (point 1) and 8.4 mm (point 10).

**Figure 15:**
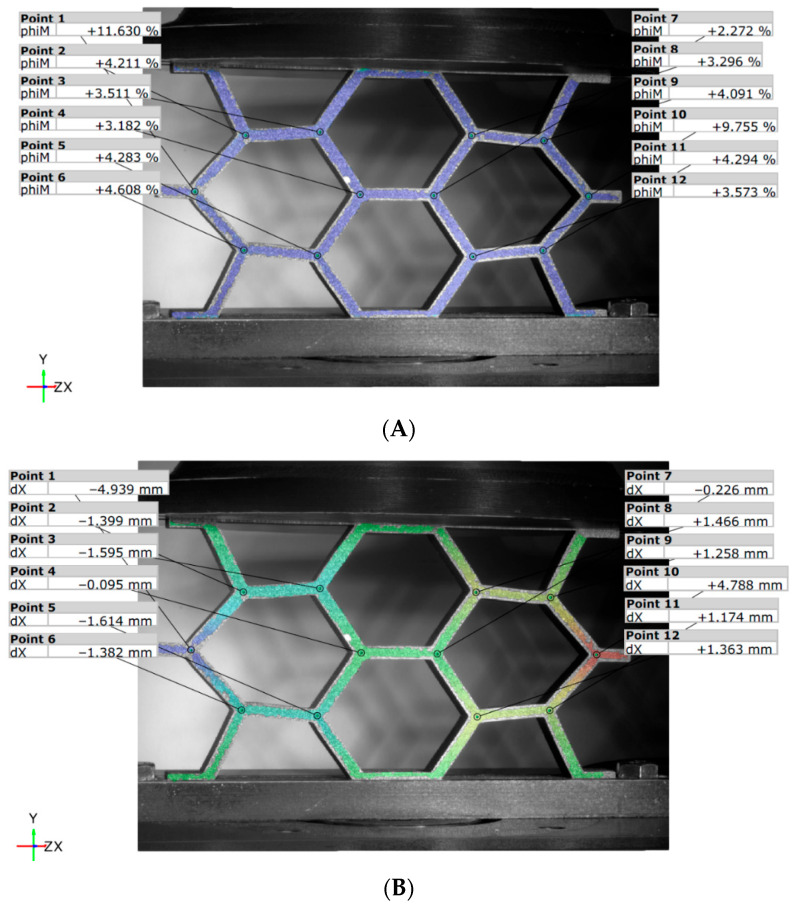
DIC results for the HCB_R panel: (**A**) von Mises strains, (**B**) displacements along X-axis.

The failure in the reinforced HCB panel occurs significantly earlier (as seen in Figure 15), resulting in lower values compared to the unreinforced version, specifically 11.6% at point 1 and 9.8% at point 10. Additionally, some notable trends can be observed. When analyzing the strains, we find that their values are relatively close to each other, ranging from 3% to 4%, with exceptions at the two previously mentioned points. In terms of displacement values, most range between 1 mm and 2 mm, with the exception of point 1, which measures 4.9 mm, and point 10, which measures 4.8 mm. It is also important to note that the displacement field is nearly symmetrical.

**Figure 16:**
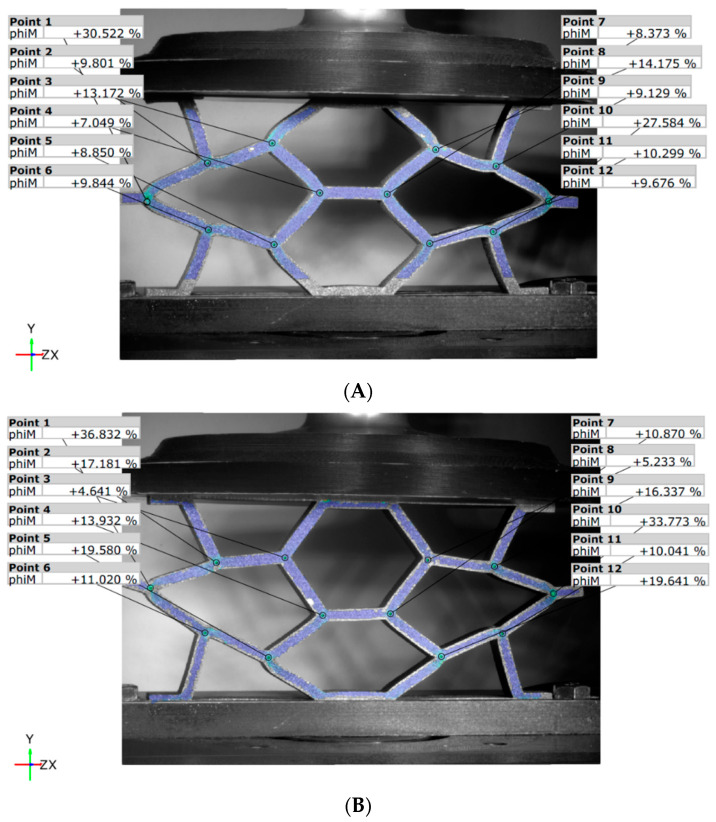
DIC von Mises strains for the HCB panel: (**A**) unreinforced; (**B**) reinforced.

At the final moment of the test, with a displacement of 15 mm (see Figure 16), it is evident that the deformation behavior of the panels is affected by the configuration used during additive manufacturing. For the unreinforced panel (Figure 16A), the strains reach maximum values of 30.5% (point 1) and 27.6% (point 10). In contrast, the reinforced panel (Figure 16B) exhibits greater rigidity, with overall greater strains, indicating that the reinforcement significantly influences the geometric behavior of the panel.

#### 3.2.2. DIC for Re-Entrant Honeycomb Panels

**Figure 17:**
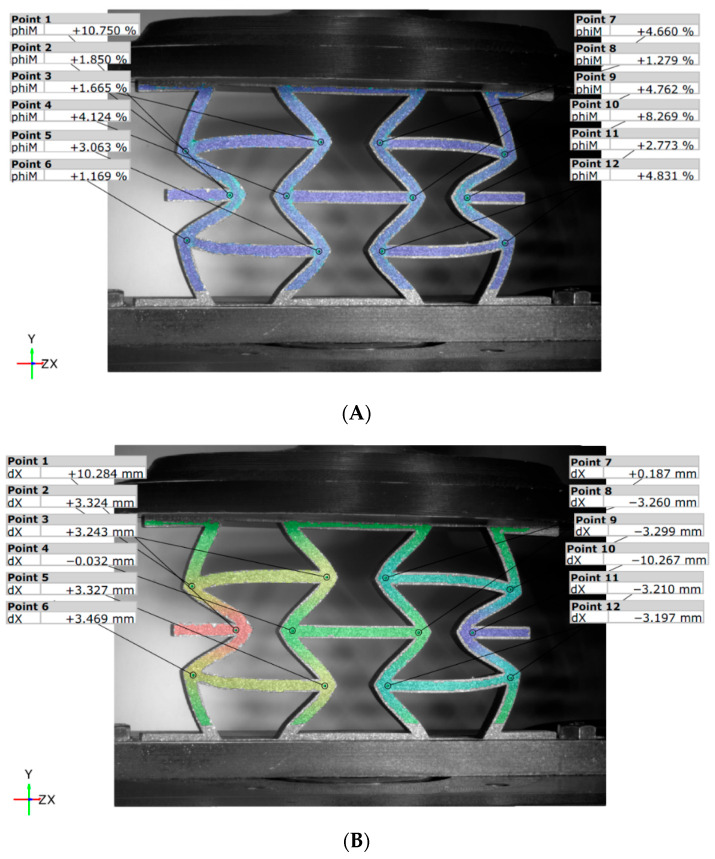
DIC results for the REN_N panel: (**A**) von Mises strains; (**B**) displacements along X-axis.

In the unreinforced re-entrant configuration REN_N (Figure 17), the maximum force leads to lower deformation values compared to the honeycomb structure, with reductions of 10.8% at point 1 and 8.3% at point 10 (Figure 17A). Additionally, the auxetic behavior of the re-entrant panel is demonstrated by the movement of points 1 and 10 along the X direction as they approach the vertical median axis by 10.3 mm for both reference points (Figure 17B).

**Figure 18:**
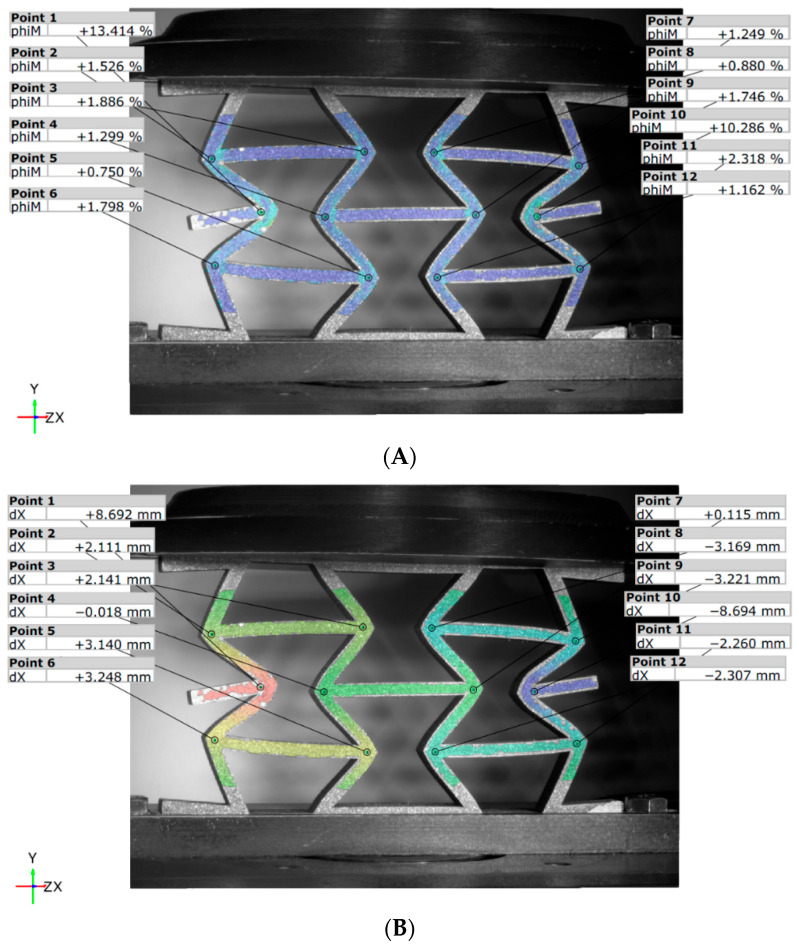
DIC results for the REN_R panel: (**A**) von Mises Strain, (**B**) displacements along X-axis.

Although the reinforced variant, REN_R (Figure 18), reaches maximum force at different moments, its strain values are similar to those of the unreinforced variant, REN_N. However, edge displacements are smaller for both variants, measuring approximately 8.7 mm (Figure 18B) compared to 10.3 mm (Figure 17B). Unlike the unreinforced topology, the reinforced panel exhibits higher stiffness and demonstrates uneven structural behavior. This leads to unbalanced deformation within the structure.

**Figure 19:**
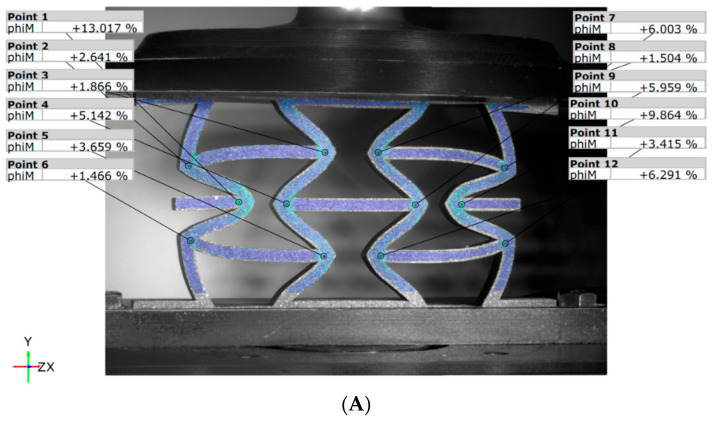

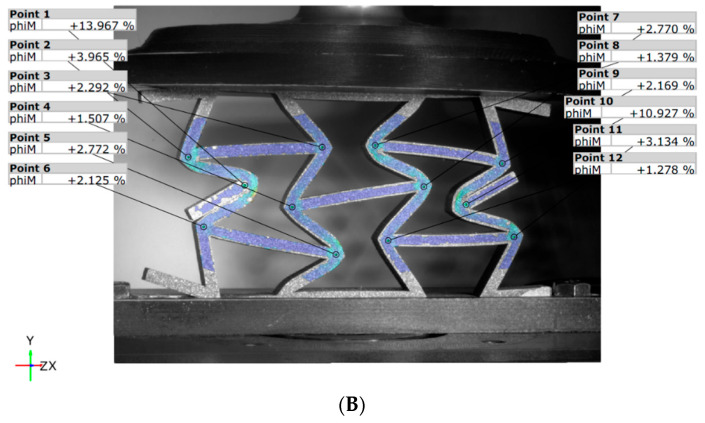
DIC von Mises strain values for the REN panels: (**A**) unreinforced; (**B**) reinforced.

At the end of the test, with a 15 mm displacement (Figure 19), the unbalanced deformation is evident in the REN_R panel (Figure 19B) when compared to the unreinforced REN_N panel (Figure 19A). The reinforced panel displays a noticeable loss of symmetry, suggesting diagonal asymmetry. This observation indicates the significant impact of the carbon fiber, as well as early signs of local fiber damage, which were also detected during the tests through acoustic emissions.

### 3.3. FEM and Experimental Analysis Results

**Figure 20:**
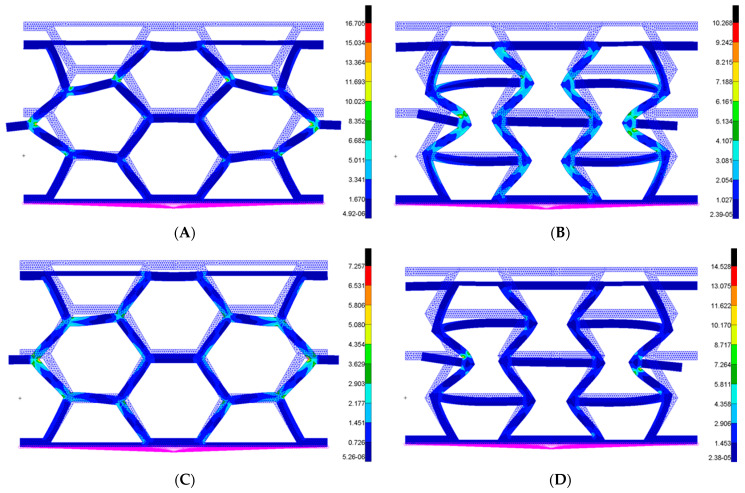
FEM analysis strain values [%] at maximum force: (**A**) HCB_N; (**B**) REN_N; (**C**) HCB_R; (**D**) REN_R.

To validate the finite element models, we compared the strain values obtained from mechanical tests analyzed using digital image correlation (DIC) with those predicted by the finite element method (FEM) simulations. Analyzing the results from all four FEM models (Figure 20), we observe a good agreement in both the maximum strain values and the edge effects. The discrepancy between the experimental and simulated results is approximately 10%, indicating that the FEM modeling approach we adopted delivers a satisfactory level of accuracy. However, we will further discuss the limitations of this modeling approach regarding the panel configurations.

**Figure 21:**
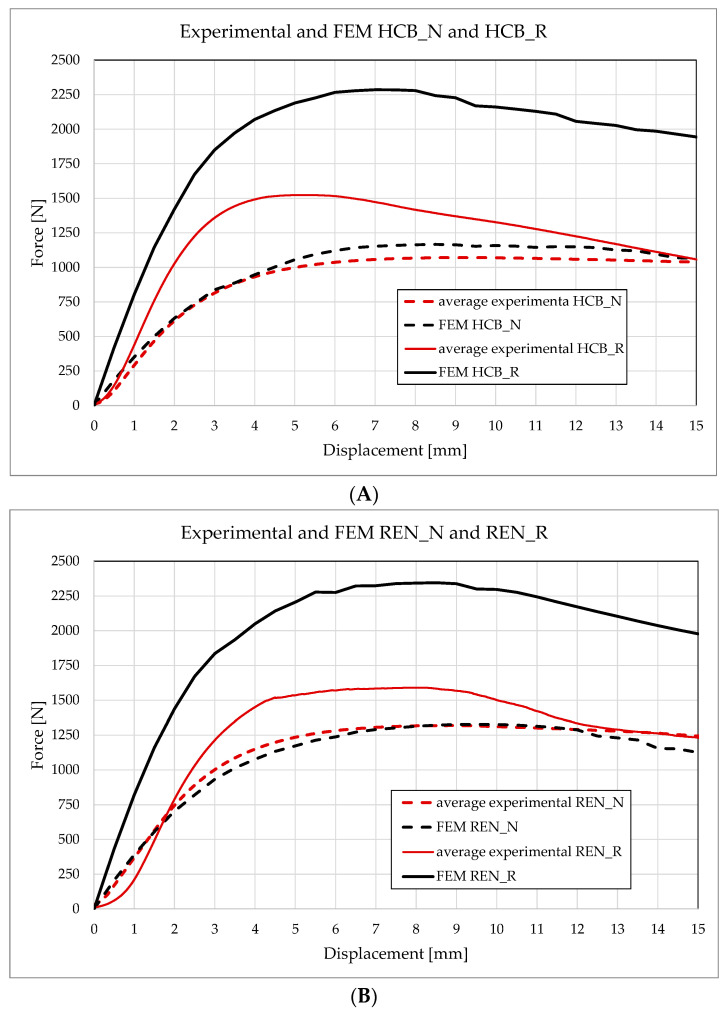
Force–displacement comparison between experimental and analytical results: (**A**) HCB; (**B**) REN.

By comparing the force–displacement plots obtained from the simulations with those measured experimentally (see Figure 21), it can be observed that the unreinforced panel configurations exhibit similar values and follow the same trend. However, this correspondence does not hold for the reinforced variants. Both the HCB_R and REN_R curves obtained through finite element modeling (FEM) significantly overestimate the average curves gathered from experimental results. The stiffness and strength observed in the simulations are notably higher. Specifically, for the HCB_R configuration, the maximum force recorded in the simulations is approximately 2250 N, while the experimental measurement is only 1500 N (refer to Figure 21A). For the REN panels, experimental and numerical maximum forces are slightly greater than those of their HCB counterparts. Nonetheless, the difference between the simulation results and the experimental results remains also substantial for REN_R configuration (see Figure 21B).

This discrepancy can be attributed to the idealization in the finite element models, emphasizing that the interface between the continuous carbon fiber and the Onyx base material is not perfect as assumed in the FEM model. The delaminations occurring between the two materials, as well as the failure behavior of the continuous carbon fiber, will be studied separately using scanning electron microscopy (SEM). Clearly, the printed reinforced material results in a suboptimal mechanical response for these reinforced panel configurations.

## 4. Discussion

As already presented, the objective was to assess the influence of fiber reinforcement on the mechanical performance and deformation response of the panel structures. In-plane compression tests were conducted to determine the stiffness, strength, and failure modes of the specimens [32,33]. Although reinforced honeycomb panels have an average load capacity that is 35% higher, they fail at a displacement that is approximately 55% smaller compared to unreinforced panels. Despite accounting for only 25% of the total number of layers and 10% of the panel’s mass, the reinforcement achieved superior strength. Re-entrant panels testing showed a 25% force increase in favor of the reinforced variant. They fail at a displacement that is 36.5% greater than that of reinforced honeycombs. This demonstrates a more compliant response while maintaining also 4.9% greater strength, indicating the superior behavior of auxetic reinforced sandwich panels. Introducing CCF reinforcement increased the load-bearing capacity and reduced localized strain concentrations without altering the overall deformation pattern.

In conclusion, the behavior of the REN_R panels demonstrated that cell geometry significantly impacts the structural performance of the panels. For the same type of reinforcement, these panels are less rigid but more compliant. Based on the comparative analysis, it can be concluded that reinforcing the cells with continuous carbon fiber has a positive structural effect, especially for re-entrant panels. However, due to their auxetic geometry, these panels exhibit a more complex response when experiencing extensive deformations.

**Figure 22:**
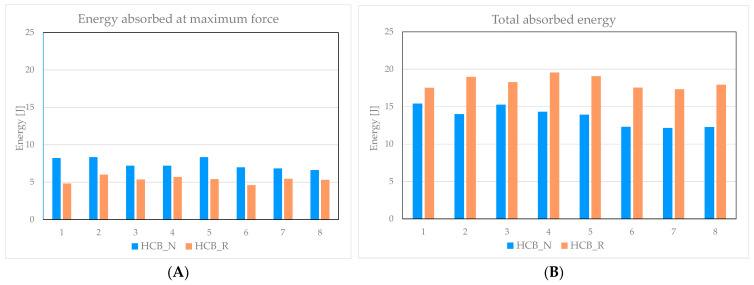
HCB panel absorbed energy: (**A**) at maximum force; (**B**) end of testing.

Another important aspect is the energy absorption capability of the two topologies, HCB and REN, in both unreinforced and reinforced variants. The energy absorbed was evaluated until the maximum force was reached and until the end of the experimental testing, which was conducted at a 15 mm displacement under compression. As shown in Figure 22A, the energy absorbed at maximum force differs by 33% between the two panel categories, favoring the HCB_N configuration. This difference mainly arises because the unreinforced panels fail at a later stage compared to the reinforced ones. However, a review of the data in Figure 22B reveals that throughout the entire test, the HCB_R panels absorb, on average, 29% more total energy than the HCB_N panels. This indicates an overall improvement in the energy absorption capacity of the reinforced configuration.

**Figure 23:**
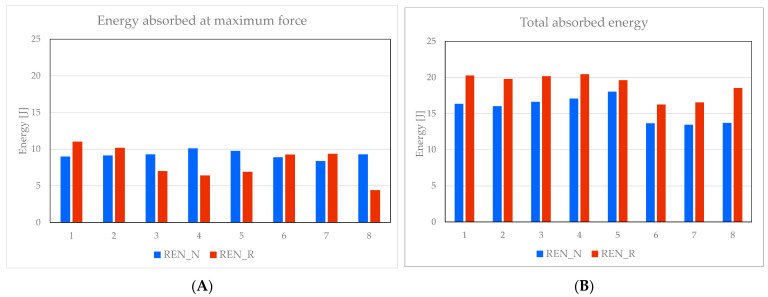
REN panels absorbed energy: (**A**) at maximum force; (**B**) end of testing.

Figure 23A indicates that, until reaching the maximum force, the competition between the REN_N and REN_R panels regarding absorbed energy is quite complex. In some cases, such as panels 3, 4, 5, and 8, the unreinforced panels absorb more energy. Conversely, in other tests, the reinforced panels (specifically panels 1, 2, 6 and 7) demonstrate greater energy absorption. By the conclusion of testing, the total absorbed energy displayed in Figure 23B clearly favors the reinforced panels across all tested samples. On average, when comparing Figure 23B to Figure 22B, the total absorbed energy of the REN_R panels surpasses that of their HCB_R counterparts.

**Figure 24:**
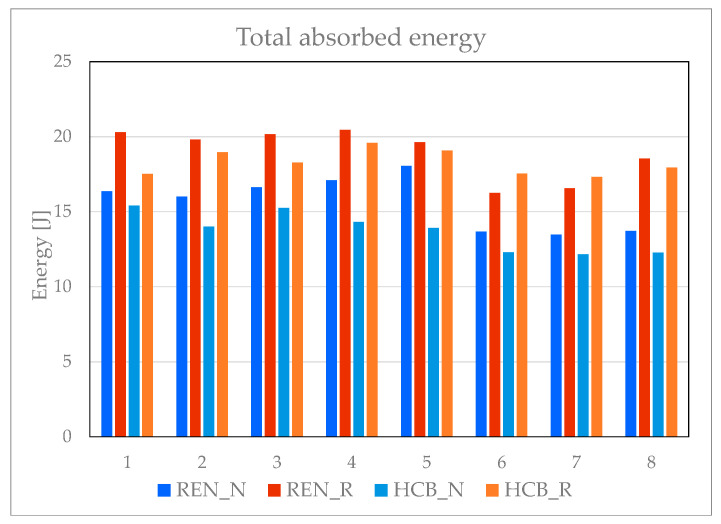
Total absorbed energy by all eight panels.

As illustrated in Figure 24, the reinforced re-entrant panels demonstrate the best overall performance in terms of energy absorption. While the difference in performance is not substantial—the average total energy absorbed by the panels is 19 J for the HCB_R panels compared to 18.3 J for the REN_R panels—the re-entrant panels offer greater compliance. This flexible behavior provides a significant advantage in various engineering applications. As for the unreinforced panels, the re-entrant configuration absorbs also more energy than the hexagonal honeycomb.

Table 13 provides a comprehensive overview of the performance of all panels by statistically quantifying the absorbed energy at maximum force and the total absorbed energy until an imposed vertical displacement of 15 mm. For each panel topology, the absorbed energies are given as average value, standard deviation, and coefficient of variation. Until the maximum registered force, the reinforced panels absorb less energy due to their rigidity, favoring the re-entrant panel with an average value of almost 51% compared to the honeycomb. By the end of testing, the reinforced panels absorbed more energy than the unreinforced panels, with 33% greater for the honeycomb panels and 21.4% for the re-entrant panels. Re-entrant panels absorb more total energy than honeycomb panels, both unreinforced and reinforced, because they are more compliant. The highest coefficient of variation is obtained for the reinforced re-entrant panels at the maximum force, at almost 26%. The other values are less than 10%.

## 5. Conclusions

We employed additive manufacturing to create hexagonal and re-entrant honeycomb panel configurations using Onyx and Onyx reinforced with continuous carbon fiber (CCF). The objective was to evaluate the impact of fiber reinforcement on the mechanical performance and deformation response of the panel structures.

The results for unreinforced panels indicate that material composition significantly impacts mechanical performance more than cell geometry. Introducing CCF reinforcement improved the load-bearing capacity and reduced localized strain concentrations; however, the overall deformation pattern remained unchanged. These results suggest that minor material enhancements can increase stiffness and strength without significantly altering strains at failure.

In conclusion, the reinforced re-entrant panel (REN_R) displays auxetic behavior and demonstrates the best overall performance in terms of maximum displacement and total energy absorbed during loading.

## Figures and Tables

**Table 1:** Properties of the used representative volumes.

Unit Cell	Mass ^1^ [g]	Cell Surface [mm^2^]	Cell Inscribed Area [mm^2^]	Mass/Area [g/mm^2^]	Relative Density [-]
Hexagonal	6.97	580	3060	0.012	0.189
Re-entrant	9.17	760			0.248

^1^ To determine the mass, the density of the Onyx base material of 1.2 g/cm^3^ (as indicated by the Markforged manufacturer) is used.

**Table 2:** Properties of Onyx material [29].

Property	Value	Unit	ASTM Standard
Density	1.2	g/cm^3^	-
Tensile Modulus	1.4	GPa	D638
Tensile Yield Strength	40.0	MPa	D638
Tensile Ultimate Strength	37.0	MPa	D638
Elongation at Break	25.0	%	D638
Flexural Strength	71.0	MPa	D7901
Flexural Modulus	3.0	GPa	D7901

**Table 3:** Properties of continuous carbon fiber (CCF) material [30].

Property	Value	Unit	ASTM Standard
Density	1.4	g/cm^3^	-
Tensile Strength	800	MPa	D3039
Tensile Modulus	60	GPa	D3039
Tensile Strain at Break	1.5	%	D3039
Flexural Strength	540	MPa	D790
Flexural Modulus	51	GPa	D790
Flexural Strain at Break	1.2	%	D790
Compressive Strength	420	MPa	D6641
Compressive Modulus	62	GPa	D6641
Compressive Strain at Break	0.7	%	D6641

**Table 4:** Information about 3D-printed unreinforced panels obtained from the slicer.

Unit Cell	Print Time per Panel [h]	No. of Panels [-]	Panel Mass [g]	Onyx Volume [cm^3^]
Hexagonal	6.65	8	34.37	29.13
Re-entrant	7.87	8	43.67	37.01

**Table 5:** Weighted mass of unreinforced additively manufactured panels.

No.	Hexagonal (HCB)		Re-Entrant (REN)	
	Mass [g]	Error [%]	Mass [g]	Error [%]
1	32.77	4.66	41.14	5.79
2	32.83	4.48	40.85	6.47
3	32.81	4.54	40.59	7.05
4	32.52	5.40	40.39	7.52
5	32.81	4.53	40.24	7.84
6	32.61	5.13	40.19	7.96
7	32.73	4.76	40.25	7.84
8	32.63	5.05	40.45	7.38
Mean Value	32.71	4.82	40.51	7.23
Slicer	34.37	-	43.67	-

**Table 6:** Information about 3D-printed reinforced panels obtained from the slicer.

Unit Cell	Print Time per Panel [h]	No. of Panels [-]	Panel Mass [g]	Onyx Volume [cm^3^]	Carbon Volume [cm^3^]
Hexagonal	7.20	8	34.90	27.95	2.61
Re-entrant	8.50	8	44.39	34.89	3.54

**Table 7:** Weighted mass of reinforced additively manufactured panels.

No.	Hexagonal (HCB)		Re-Entrant (REN)	
	Mass [g]	Error [%]	Mass [g]	Error [%]
1	32.71	6.27	41.44	6.64
2	32.78	6.08	41.56	6.38
3	32.80	6.03	41.41	6.70
4	32.67	6.40	41.45	6.63
5	32.44	7.04	41.45	6.63
6	32.56	6.70	41.41	6.72
7	32.70	6.31	40.88	7.90
8	32.89	5.75	41.83	5.76
Mean Value	32.69	6.32	41.43	6.67
Slicer	34.90	-	44.39	-

**Table 8:** Results of tensile tests for the two material configurations.

Property	Unreinforced (Onyx)	Reinforced (Onyx + CCF)
Modulus of Elasticity	800 MPa	2700 MPa
Tensile Strength	35 MPa	135 MPa
Yield Strength	20 MPa	-
Strain at break	40%	4.8%

**Table 9:** Maximum force values for HCB_N panels.

Panel	Maximum Force [N]	Displacement [mm]
HCB_N_1	1210.26	8.96
HCB_N_2	1106.13	9.58
HCB_N_3	1183.28	8.08
HCB_N_4	1133.64	8.39
HCB_N_5	1092.05	9.81
HCB_N_6	955.54	9.37
HCB_N_7	939.78	9.30
HCB_N_8	949.74	8.96
Average	1071.30	9.06

**Table 10:** Maximum force values for REN_N panels.

Panel	Maximum Force [N]	Displacement [mm]
REN_N_1	1319.10	8.90
REN_N_2	1277.32	9.56
REN_N_3	1383.83	8.83
REN_N_4	1332.94	9.69
REN_N_5	1514.15	8.39
REN_N_6	1057.02	10.46
REN_N_7	1048.48	10.09
REN_N_8	1063.10	10.82
Average	1249.49	9.59

**Table 11:** Maximum force values for HCB_R panels.

Panel	Maximum Force [N]	Displacement [mm]
HCB_R_1	1500.42	4.78
HCB_R_2	1531.07	5.48
HCB_R_3	1476.06	5.18
HCB_R_4	1663.15	5.08
HCB_R_5	1626.10	5.10
HCB_R_6	1466.42	4.59
HCB_R_7	1466.96	5.42
HCB_R_8	1486.77	5.08
Average	1527.12	5.09

**Table 12:** Maximum force values for REN_R panels.

Panel	Maximum Force [N]	Displacement [mm]
REN_R_1	1687.69	8.73
REN_R_2	1683.21	7.60
REN_R_3	1695.14	6.31
REN_R_4	1726.41	5.91
REN_R_5	1737.96	5.67
REN_R_6	1403.20	8.29
REN_R_7	1376.35	8.54
REN_R_8	1510.44	4.56
Average	1602.55	6.95

**Table 13:** Absorbed energy at maximum force and total absorbed energy for all panels.

Type of Sandwich Panel	Energy Absorbed at Maximum Force [J]			Total Absorbed Energy [J]		
	Average	Standard Deviation	Coefficient of Variation [%]	Average	Standard Deviation	Coefficient of Variation [%]
REN_N	9.22	0.49	5.34	15.62	1.65	10.58
REN_R	8.06	2.09	25.93	18.96	1.58	8.31
HCB_N	7.46	0.67	8.97	13.70	1.24	9.03
HCB_R	5.34	0.42	7.83	18.27	0.78	4.31

## Data Availability

The data presented in this study are available from the corresponding author upon request. The data are part of an unfinished Ph.D. thesis.
